# Infraglenoid Muscle as an Anatomic Variation of the Anterior Rotator Cuff

**DOI:** 10.1155/2019/6938252

**Published:** 2019-02-10

**Authors:** Gwan Bum Lee, Erica Kholinne, Jae-Man Kwak, Yucheng Sun, Adel Mohammaed Alhazmi, In-Ho Jeon

**Affiliations:** ^1^Department of Orthopedic Surgery, Asan Medical Center, University of Ulsan College of Medicine, Seoul, Republic of Korea; ^2^Department of Orthopedic Surgery, St. Carolus Hospital, Jakarta, Indonesia

## Abstract

**Case:**

We report a patient with osteoarthritis of the shoulder joint who underwent reverse shoulder arthroplasty and showed anatomical variation in the subscapularis muscle (SM). A variation of the rotator cuff originating from the anteroinferior aspect of the glenoid was separated from the SM by a septum and was named infraglenoid muscle (IGM).

**Conclusion:**

We suggest that the IGM may serve as an additional dynamic stabilizer for external rotation. Studying this particular muscular variation would lead to better understanding of the anatomical structures around the glenoid cavity.

## 1. Introduction

Among the four major muscles of the rotator cuff, the subscapularis muscle (SM) is the largest and strongest. SM allows internal rotation of the humerus, provides anterior stability of the shoulder, and balances the force couples of the glenohumeral joint [1]. A number of accessory muscle slips connected with the SM have been reported as variations of SM [2–6].

In 2012, anatomical variation in the anterior-inferior rotator cuff was found through the dissection course of 67 shoulders and was named “infraglenoid muscle (IGM)” in order to distinguish it from accessory muscle slips [7].

We report the case of a patient with osteoarthritis of the shoulder joint who underwent anatomic shoulder arthroplasty at our hospital. An incidental finding of IGM was found intraoperatively. The patient was informed that data concerning the case would be submitted for publication, and she provided consent.

## 2. Case Report

**Figure 1:**
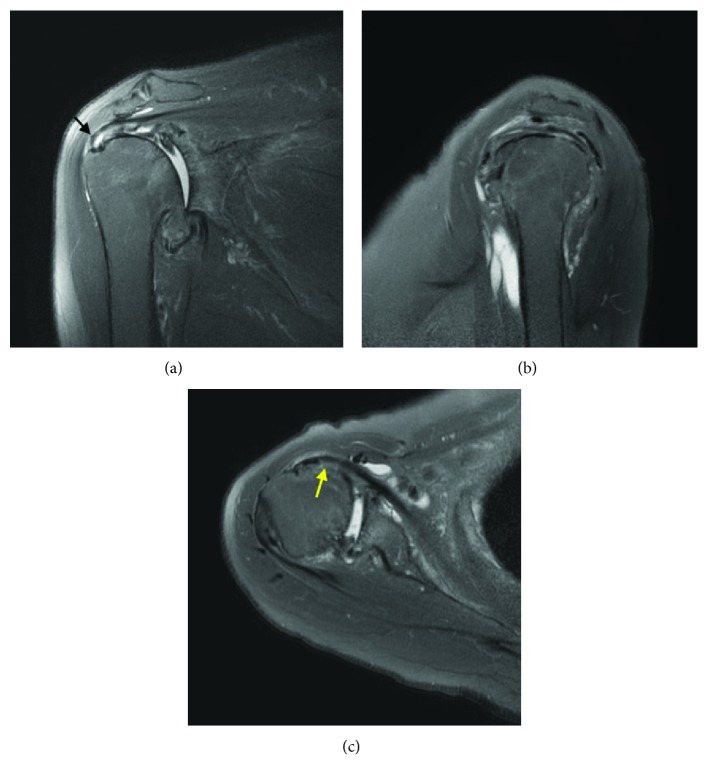
MRI T2-weighted of the right shoulder: coronal oblique view (a) showing a small size full thickness supraspinatus tear (black arrow), sagittal view (b), and axial view (c) showing tendinosis of the subscapularis muscle (yellow arrow).

A 70-year-old female with a history of three years of right shoulder pain presented with limitation in both active and passive ranges of motion. Upon physical examination, Jobe's test and Hawkins test both showed positive results, indicating dysfunction of the rotator cuff and impingement in the shoulder joint, respectively. Plain radiograph showed joint space narrowing with bone deformity, sclerotic change, and subchondral cyst formation in the glenohumeral joint; the patient was thus diagnosed as advanced osteoarthritis. MRI evaluation revealed an approximately 1 cm tear in the rotator cuff, specifically in the supraspinatus muscle on its articular side, as well as tendinosis and subtle muscular signal change in distal subscapularis (Figure 1). The patient underwent an anatomic shoulder arthroplasty considering the functional deltoid muscle.

### 2.1. Intraoperative Findings

**Figure 2:**
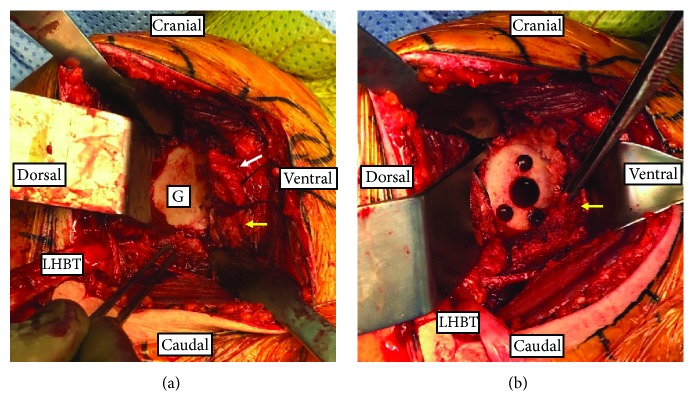
Intraoperative picture showing infraglenoid muscle (yellow arrow) originating from the anteroinferior aspect of glenoid (G), separate from subscapularis muscle (white arrow): (a) prior to glenoid preparation and (b) after glenoid reaming. G: glenoid; LHBT: long head of biceps tendon (transected).

The deltopectoral approach was used for the surgery. Upon blunt dissection to the subdeltoid and subacromial spaces, a superior portion of the pectoralis major muscle was released. The subscapularis tendon was released from its insertion site followed by capsulotomy. A distinct muscular structure was found at the anterior-inferior aspect of the glenoid rim (Figure 2), which was not determined as part of the glenoid labrum. This muscle was carefully tagged and dissected near its origin site to allow glenoid reaming. A routine anatomic total shoulder arthroplasty was carried out afterwards. The muscle was repaired along with the SM. No major complications were found. The patient was discharged at seven days postoperation.

## 3. Discussion

The IGM has been referred to by a range of different names including subscapulo-capsular muscle, subscapulo-humeral muscle, subscapularis minor muscle, infraspinalis secundus, subscapularis secundus, axillary slip of the subscapularis, and accessory subscapularis-teres-latissimus muscle, thereby causing difficulties in communication [2, 8]. In 2012, Staniek and Brenner reported 43 IGMs from 67 shoulders, an incidence rate of 64% [7]. IGM originated from the lateral border of the scapula in the so-called “marginal axillary groove” and inserted to the crest of the lesser tubercle or lesser tubercle itself. The origin site reported in the previous study coincided with that of the muscle found in our case, although we could not identify the insertion site. In the aforementioned study, the IGM was separated from the SM by a septum as part of subscapularis fascia, which was in accordance with our case.

Staniek and Brenner reported that the IGMs in 19 of 43 cases were innervated by the axillary nerve. Taking into account the origin and insertion of IGM, the authors discussed that the function of this muscle is distinct from the SM and that IGM would carry an adjunctive role in adduction together with the rotator cuff. Yoshinaga et al. reported that the so-called accessory SM is innervated by fibers of the dorsal element of C7 immediately cranial to the thoracodorsal nerve, indicating that the accessory SM might be close to the formation of the latissimus dorsi muscle in its derivation rather than the SM [2]. We are in the opinion that this accessory SM is the same structure with IGM.

Several studies reported a relationship between the accessory muscle and nervous structures, especially the axillary nerve. Breisch reported that the accessory SM, along with the SM, forms the myotendinous tunnel through which the axillary nerve and inferior subscapular nerve pass and may be clinically associated with entrapment neuropathy [3]. Pires et al. reported that the accessory SM may cause axillary nerve entrapment in the quadrangular space [8], possibly due to the fact that the accessory muscle possesses a large fleshy portion.

The clinical role of the IGM is still under debate, with a previous study reporting that there are no consistent findings on this muscle [4, 6, 8]. We suggest that the IGM might serve as an additional dynamic stabilizer for external rotation and that it is a remnant anatomical structure which failed to fully differentiate as a part of the SM. An alteration of mesenchymal differentiation may play a role in this anatomic variation. The incidence rate of IGM is higher than expected, as reported by Staniek and Brenner; therefore, gaining knowledge on this anatomic variation is important, especially when approaching the glenoid cavity. Surgeons may encounter IGM during procedures that expose the glenoid cavity or the lateral aspect of the scapula. By understanding IGM better, we can further grasp the anatomical structures around the glenoid cavity, especially the axillary nerve. IGM may also serve as a landmark for orientation of the glenoid cavity and thus should be preserved during surgical procedures. Further studies are needed to uncover the exact functions of the IGM.

## 4. Conclusion

Reports on the existence of infraglenoid muscle as an anatomic variation of the anterior-inferior rotator cuff are very scarce. This case report described the IGM as a supernumerary muscle to the rotator cuff. The IGM may be considered as an additional stabilizer of the anterior shoulder dynamics.

## References

[1] Longo U. G., Berton A., Marinozzi A., Maffulli N., Denaro V. (2012). Subscapularis tears. *Medicine and Sport Science*.

[2] Yoshinaga K., Kawai K., Tanii I., Imaizumi K., Kodama K. (2008). Nerve fiber analysis on the so-called accessory subscapularis muscle and its morphological significance. *Anatomical Science International*.

[3] Breisch E. A. (1986). A rare human variation: the relationship of the axillary and inferior subscapular nerves to an accessory subscapularis muscle. *The Anatomical Record*.

[4] Kameda Y. (1976). An anomalous muscle (accessory subscapularis-teres-latissimus muscle) in the axilla penetrating the brachial plexus in man. *Acta Anatomica*.

[5] A-F L. D. (1897). *Traité des variations du système musculaire de l'homme et de leur signification au point de vue de l'anthropologie zoologique*.

[6] Macalister A. (1867). Notes on an instance of irregularity in the muscles around the shoulder joint. *Journal of Anatomy and Physiology*.

[7] Staniek M., Brenner E. (2012). Variations in the anatomy of the anterior-inferior rotator cuff: the “infraglenoid muscle”. *Annals of Anatomy*.

[8] Pires L. A. S., Souza C. F. C., Teixeira A. R., Leite T. F. O., Babinski M. A., Chagas C. A. A. (2017). Accessory subscapularis muscle - a forgotten variation?. *Morphologie*.

